# Laser-induced microinjury of the corneal basal epithelium and imaging of resident macrophage responses in a live, whole-eye preparation

**DOI:** 10.3389/fimmu.2023.1050594

**Published:** 2023-02-06

**Authors:** Sebastian M. D. Gulka, Brent Gowen, Anastasia M. Litke, Kerry R. Delaney, Robert L. Chow

**Affiliations:** ^1^ Department of Biology, University of Victoria, Victoria, BC, Canada; ^2^ University of Illinois College of Medicine, Chicago, IL, United States

**Keywords:** cornea, wound healing, macrophage, live-cell imaging, confocal laser scanning electron microscope, epithelium

## Abstract

The corneal epithelium is continuously subjected to external stimuli that results in varying degrees of cellular damage. The use of live-cell imaging approaches has facilitated understanding of the cellular and molecular mechanisms underlying the corneal epithelial wound healing process. Here, we describe a live, *ex vivo*, whole-eye approach using laser scanning confocal microscopy to simultaneously induce and visualize short-term cellular responses following microdamage to the corneal epithelium. Live-cell imaging of corneal cell layers was enabled using the lipophilic fluorescent dyes, SGC5 or FM4-64, which, when injected into the anterior chamber of enucleated eyes, readily penetrated and labelled cell membranes. Necrotic microdamage to a defined region (30 μm x 30 μm) through the central plane of the corneal basal epithelium was induced by continuously scanning for at least one minute using high laser power and was dependent on the presence of lipophilic fluorescent dye. This whole-mount live-cell imaging and microdamage approach was used to examine the behavior of *Cx3cr1:GFP*-expressing resident corneal stromal macrophages (RCSMs). In undamaged corneas, RCSMs remained stationary, but exhibited a constant extension and retraction of short (~5 μm) semicircular, pseudopodia-like processes reminiscent of what has previously been reported in corneal dendritic cells. Within minutes of microdamage, nearby anterior RCSMs became highly polarized and extended projections towards the damaged region. The extension of the processes plateaued after about 30 minutes and remained stable over the course of 2-3 hours of imaging. Retrospective immunolabeling showed that these responding RCSMs were MHC class II+. This study adds to existing knowledge of immune cell behavior in response to corneal damage and introduces a simple corneal epithelial microdamage and wound healing paradigm.

## Introduction

The corneal epithelium is the outermost, multi-layered region of the cornea and first point of contact for many forms of corneal damage caused by physical and chemical injury, as well as pathological conditions (1–4). Maintenance of corneal health and transparency is essential for proper vision and depends on a rapid response of the corneal epithelium to injury in order to re-establish its barrier function to prevent entry of noxious environmental factors (5, 6). Similar to wound healing in the skin (7), corneal epithelial wound healing is mediated by a complex set of processes that involve cell migration, proliferation, re-stratification, matrix deposition and tissue remodeling (2, 6, 8). These processes are regulated by the signaling and cross-talk of several growth factors and cytokines, and other receptors (e.g. purinergic receptors, Toll-like receptor 4) that act on the ERK, MAP kinase, and/or NF-kB pathways, as well as Rho-family GTPase and extracellular protease (e.g. matrix metalloproteinases) activity (2).

Several *in vitro* and *in vivo* experimental approaches have been developed to study various aspects of corneal epithelial wound healing such as re-epithelialization and cell proliferation rates, re-innervation and immune system responses (reviewed in (9)). *In vivo*, different approaches have been developed to remove specific parts of the corneal epithelium. For example, epithelial debridement performed using a blunted razor leads to the removal of all corneal epithelial layers. Removing the entire epithelium can also be performed using a rotating diamond burr can also lead to keratectomy (i.e., removal of the basement membrane). Alternatively, removal of only the superficial epithelium, leaving the basal epithelium intact, can be achieved by touching a dry piece of filter paper to the corneal surface. Other approaches, such as ex vivo, organ culture and *in vitro* 2D culture of primary cultures of corneal epithelial cell have also been useful for examining proliferation and migration. These approaches have the benefit of enabling one to perform experiments on human donor corneal tissue. They also enable experiments that would be much harder to perform *in vivo*, such as examining the effects of growth factors, blocking antibodies and drugs, or transfection of expression constructs.

Since the initial finding of a resident population of MHC(-) dendritic cells in the peripheral cornea (10), subsequent studies have shown that the cornea is able to host to a number of different leukocytes. This includes a resident population of cells that are differentially distributed throughout the peripheral and central cornea and include dendritic/Langerhans cells (LCs), mast cells (MCs), macrophages, γδ T lymphocytes, and innate lymphoid cells (reviewed in (11–13)). In addition, corneal wound healing and ensuing inflammation response can lead to the recruitment of circulating leukocytes such as neutrophils, macrophages and dendritic cells (13–15). Tight control of the immune system and inflammation is essential for striking a balance between maintaining corneal transparency and enabling an effective and appropriate wound healing response (1, 6, 8, 16–20).

The cornea’s innate transparency and external ocular position make it well-suited for live-imaging. This has enabled live-imaging of fluorescent protein-expressing leukocytes in corneal wholemount explant cultures to various forms of external stimulation and damage (21–25).﻿ In the undamaged cornea, dendritic cells exhibited a stationary but ﻿regular, repetitive extension and retraction of dendritic processes (21, 22) that has been termed “dendrite surveillance extension and retraction cycling habitude” (dSEARCH) (21). Pinpoint thermal injuries delivered to individual GFP+ dendritic cells resulted in neighboring (within 100 μm) dendritic cells displaying with augmented activity as well as lateral movement (21). ﻿Stimulation of a 1 mm dimeter region of the central cornea using either silver nitrate injury, injection with lipopolysaccharide injection, or microspheres, resulted in the processes of ﻿*CD11c^eYFP^* + dendritic cells (which also labels a population of sub-epithelial macrophages) ﻿orienting toward the stimulus but with minimal migration (22). In a live-mouse model of contact lens wear, imaging of corneas from *Lys2^GFP^* + (myeloid-derived) and *CD11c-YFP* reporter mouse strains revealed changes in the number and distribution of corneal leukocytes as well as neutrophil recruitment over the course of 1 to 13 days (23).

Here, we have developed an *ex vivo*, whole-eye, live-imaging approach that utilizes lipophilic fluorescent dye labelling to visualize corneal cell membranes using laser scanning confocal microscopy. Laser scanning at high power within the central plane of the basal epithelium led to robust, dye-dependent microdamage of the epithelium within a precisely-defined area. This microdamage approach enabled real-time, high-resolution visualization of resident central corneal macrophages in *CX3CR1^+/GFP^* eyes and revealed rapid responses characterized by cell polarization and the formation of pseudopodia-like processes extending towards the site of damage. This study adds to our existing knowledge of monocyte behavior in response to corneal damage in live-cell preparations and introduces a simple corneal epithelial microdamage and wound healing paradigm.

## Methods

### Mice

All experiments were performed following approval by the University of Victoria Animal Care Committee in accordance with guidelines set by the Canadian Council for Animal Care. Mice on a 129S1 genetic background mice were used for laser-damage assay testing. *CX3CR1^+/GFP^* mice (26), were used for live and fixed imaging of *Cx3cr1*-expressing immune cells. All animals were maintained on a 12-hour light/dark cycle and were 3-6 months of age.

### Anterior chamber injection

Mice were euthanized by inducing deep anesthesia with isoflurane followed by cervical dislocation. Eyes were enucleated and a pilot hole in the outer edge of the cornea was made by gently inserting a 30-gauge beveled insulin syringe at the edge of the cornea as previously described (27). A 50 µl Hamilton glass syringe (model 705 RN SYR) with a 31-gauge, beveled needle was loaded with 4 μl of dye. Before injection into the anterior chamber, the plunger was first depressed until a small bead formed on the end of the needle to ensure there were no air bubbles, then the needle tip was inserted into the pilot hole and slowly injected until the cornea was visibly filled with dye at which point the injection was stopped. Injected dyes include: 1 mM Alexa 647 hydrazide (Cat. number A20502, Thermo Fisher, Waltham, MA), 1 mM of SGC5 (Cat. number 70057, Biotium Inc., Fremont, CA) and 1 mM FM4-64 (Cat. number T13320, Thermo Fisher, Waltham, MA). Eyes were imaged within five minutes post injection.

### Laser scanning confocal microscopy

For live whole-mount imaging, enucleated mouse eyes were placed into brain buffer (BB; 135 mM sodium chloride, 2.7 mM potassium chloride, 2 mM calcium chloride, 10 mM HEPES buffer, pH 7.4 and 2 mM magnesium chloride)-filled 4 mm diameter wells made by pouring a small amount of Sylgard (Dow) into a 35mm petri dish and embedding the cut ~5mm bottom of a 0.6 mL centrifuge tube into the Sylgard before it hardened. Eyes were imaged at room temperature (~22 C°) using a 60x water dipping lens (NA 1.0, WD 2.0 mm, Nikon NIR APO 60x) on a Nikon C2 confocal microscope. For live images were obtained, z-stacks were set 1.0-1.5 µm apart. Live time-lapse images were obtained at a pixel density of 512x512 with a pixel dwell time of 4.8 µS using laser power set at or below 2% (18 μW).

### Basal epithelial cell microdamage

**Figure 1:**
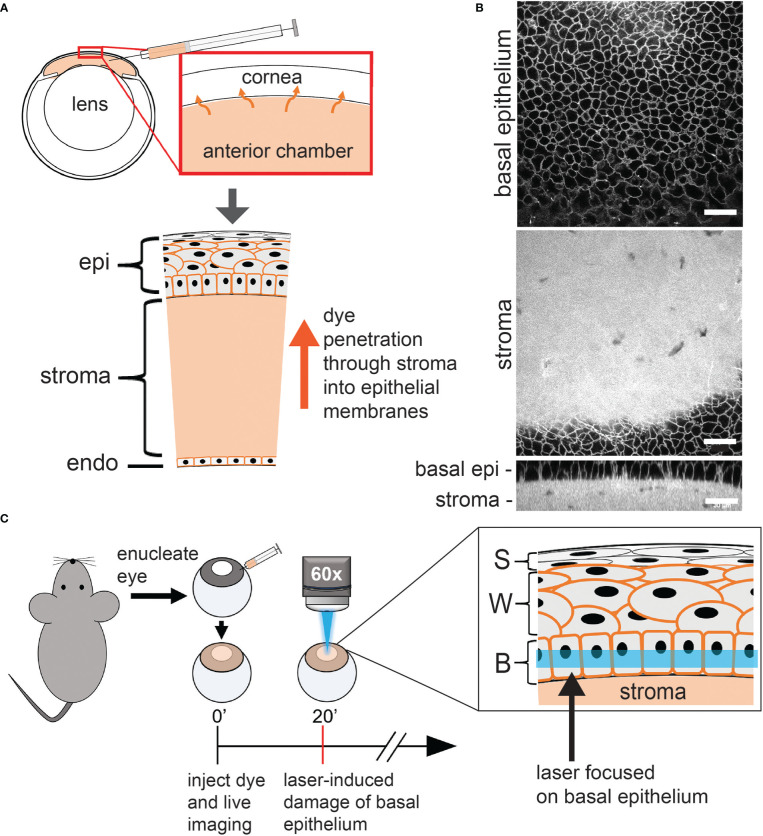
Overview of the confocal laser scanning microscopy induction of basal epithelium microdamage. **(A)** Schematic of cornea and anterior chamber injection. Alexa 647 hydrazide and either SGC5 or FM4-64 (orange in diagram) injected into the anterior chamber, readily diffuses into the cell membranes of all corneal layer. SGC5 and FM4-64 labelled cell membranes while Alexa647 hydrazide was confined to the stroma. **(B)** Confocal microscope imaging of SGC5 labelling in cell membranes of the corneal basal epithelium and stroma. **(C)** Corneal basal epithelium laser-damage protocol. Eyes from euthanized mice were enucleated, injected with dye, bathed in brain buffer and a defined region of cornea imaged at high laser power for 1-10 minutes. epi, corneal epithelium; (b) epi, basal epithelium; S, superficial epithelium; W, wing cells; B, basal epithelium. Scale bar in B = 30µm.

Dye-injected eyes (as described above, **Figure 1A**) were prepared as above and focused ~6 µm anterior to the basal epithelial-stromal interface. The 488 nm or 405 nm laser lines were used to damage eyes injected with SGC5, and the 561 nm laser of the confocal microscope for eyes injected with FM4-64. Damage was typically induced by zooming in 6x to scan a region of approximately 30 µm x 30 µm continuously for a minimum 60 seconds using different laser power settings as described in the results section. After induction was complete, the field of view was returned to a zoom factor of 1x and imaged using a laser power of 2% (18 μW) or lower for up to 2.5 hours. For experiments on eyes from *Cx3cr1^+/GFP^* mice, areas devoid of GFP+ cells anywhere along the Z-axis of the scanned region were selected for microdamage to avoid the inclusion of GFP+ macrophages that may have been subjected to secondary, laser-induced damage activation.

### Image analysis and quantification of cell dynamics

Images were analyzed using the Fiji suite of ImageJ (28). This included 3D drift correction for time-lapse images, pixel intensity adjustments, background subtraction, and distance measurements. ImageJ was used to quantify cell dynamics of both steady-state and injury-responding cells. Time-lapse images were first corrected using plugins>registration>correct 3D drift. A maximum projection of the cells of interest was generated. When measuring length change of cell processes, the first timepoint captured was used as the reference position. A perpendicular line was drawn through the base of the process in the initial reference position and a spot was marked in the middle of that line as the origin to measure from. Lengths were measured from the reference point of the specified cell process to the furthest edge of the cell process at the next timepoint. Distance of “*responding*” or “*non-responding*” cells to the site of microdamage was defined as the distance from nearest edge of the cell to the nearest point of the damaged area. “*Responding*” cells were defined as those in which at least one pseudopodia-like projection exhibited a sustained (>30 minutes) increase in length (> 10 μm) towards the site of damage as measured 1-hour after damage protocol was performed. Distances were calculated in ImageJ by first measuring distances in the XY direction and in the Z distance and then calculating the hypotenuse of those lengths to find the true distance. A two tailed unpaired T test was performed on the distance means.

### Electron microscopy

Corneas were fixed in PFA/GLUT (3% paraformaldehyde and 3% glutaraldehyde in 1xPBS) for 10 minutes and corneas were dissected away from the eye. A second 40-minute fix in PFA/GLU was done to the isolated cornea. The fixed cornea was washed twice in 1x PBS then stored in 1x PBS at 4°C prior to EM processing. The cornea was then prepared for standard Epon embedding involving osmium tetroxide, dehydration in ethanol series, and embedding into Epon.

### Immunolabeling

After confocal imaging, corneas were fixed in 4% PFA for 30 minutes, washed in PBS (phosphate-buffered saline; 8% NaCl, 0.2% KCl, 1.6% Na2HPO4, 0.24% KH2PO4), then stored in PBS at 4°C until immunostaining. The cornea was blocked in a solution containing 10% horse serum and 0.3% triton in PBS (PBST-HS) for 12 hours, then incubated with the 1° antibodies overnight at 4°C while being slowly rotated. The corneas were then washed in PBST-HS three times for a total of 10 hours then incubated with the 2° antibodies as per the 1°’s, washed for four hours, then given a final wash in PBS for 5 minutes before mounting with Immumount (Thermo Fisher, Waltham, MA). 1° Antibodies: goat anti-GFP (1:500, Abcam, Cat. No. ab6673, Eugene, OR), rat anti-MHCII Monoclonal Antibody (M5/114.15.2), eBioscience (I-A/I-E) (1:500 Thermo Fisher, Cat. No. 14-5321-82, Waltham, MA). 2° antibodies: donkey anti-goat Alexa Fluor 488 (Thermo Fisher, Cat. No. A-11055), goat anti-rat Alexa Fluor 555 (Thermo Fisher, Cat. No. A78945) both used at 1:500.

## Results

### Live whole-mount laser scanning confocal imaging of the cornea and induction of basal epithelium microdamage

**Figure 2:**
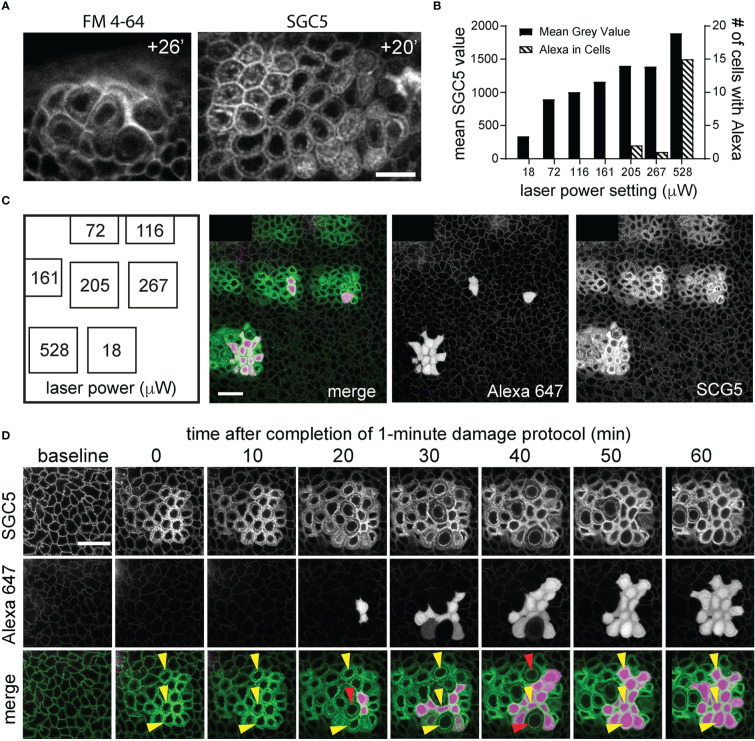
Laser power titration and Alexa 647 hydrazide dye cellular uptake following corneal basal epithelial cell microdamage. **(A)** Imaging of the basal epithelium injected with FM4-64 or SGC5 lipophilic membrane dyes at times (in minutes) following confocal laser scanning microscopy-induced basal epithelial cell microdamage. **(B, C)** Results from a single experiment in which regions of the corneal basal epithelial were scanned for 1 minute with the 488 nm laser line set at the power indicated on the x-axis and imaged 1 hour later at low laser power (488 nm at 18 µW, 640 nm at <2% laser power). The graph in **(B)** shows quantification of the single experiment performed in panel **(C)** plotting the mean SGC5 fluorescence levels above background and number of cells with Alexa 647 hydrazide cellular uptake for each of the scanned regions. **(D)** Time-course of corneal basal epithelium from panel **(C)** that was scanned at 528 μW for 1 minute at 488 nm. SGC5 dye internalization is observed immediately following the 1-minute 528 μW laser power scan and progressively increases over time as does cell swelling. Alexa 647 dye uptake was first observed 20 minutes after the high-power scan in 2 cells and increased to approximately 75% of the scanned cells after 1 hour. Three different cells are labelled with yellow arrowheads and show maximal cell swelling at time points indicated by the red arrowheads and followed at the next time point by cell contraction and Alexa 647 hydrazide uptake. Scale bars in **(A, C, D)** = 15 µm.

In order to visualize corneal cell types in live wholemount preparations, we injected the styrl lipophilic dye SGC5 (29) into the anterior chamber of enucleated eyes as described previously by our lab (**Figure 1A**) (27). When injected, SGC5 readily penetrated and diffused through the corneal endothelium, stroma and epithelial layers, and labelled cell membranes (**Figure 1B** and data not shown). Confocal imaging of the central-most region using a water-dipping 60x objective and the 488 nm laser line at 2% power (18 μW) allowed stable visualization of all corneal cell membranes without any visible change for the duration of the imaging experiments which typically lasted between 2-3 hours (**Figure 2C** data not shown). In uninjected eyes bathed in SGC5, the endothelium, stroma, basal epithelium or wing cell membranes remained unlabeled, likely due to the impenetrable, barrier function of the anterior-most superficial layers of the corneal epithelium (30).

**Figure 3:**
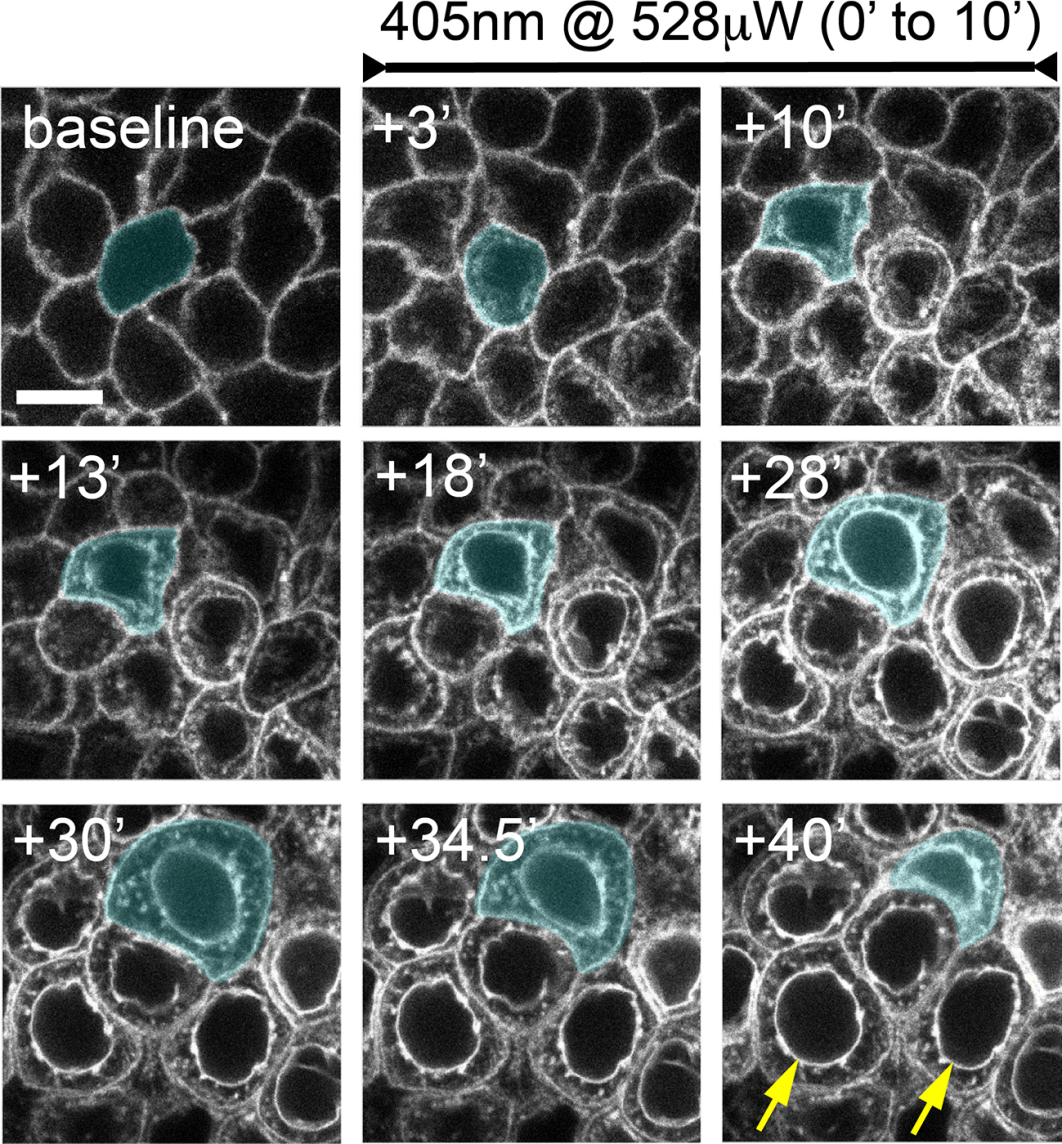
Confocal laser scanning microscopy-induced basal epithelial cell microdamage. Corneas were imaged for 30 minutes using the 488 nm laser line at low-power (18 μW) which allowed for stable imaging of SGC5 dye-labelled cell membranes. The basal epithelium was then co-scanned with a 405 nm laser (528 μW) for 10 minutes. Within 3 minutes, fluorescence was now observed within cells, and membrane fluorescence increased. Laser-damaged epithelial cells began swelling followed by contraction within 40 minutes of the onset of the 405 nm laser. A cell undergoing swelling (+10’ to +30’) and contraction (+34.5’ to +40’) is shaded blue. The yellow arrows in the bottom right panel indicate presumptive nuclear membrane labeling. Times indicated are in minutes following onset of 405 nm laser scanning. Scale bar = 7.5µm.

Serendipitously, we observed that SGC5-injected corneas imaged using the 488 nm or 405 nm laser lines set at or above 72 μW or 528 μW, respectively, and focused on a single plane set 6 µm above the basal epithelial-stromal interface (**Figure 1C**), led to rapid changes consistent with cellular damage. The first change was an increase in SGC5 cellular fluorescence due to the apparent internalization into basal epithelial cells. This increase was observed almost immediately after onset of high-power scanning and led to the gradual labeling of internal cellular structures including the presumptive nuclear membrane (**Figures 3**, yellow arrows; **2D**). After 10 minutes of scanning, cell swelling was observed often followed by contraction (see cyan colored cell in **Figure 3**). Similar responses were also observed with as little as 1 minute of laser scanning and showed a graded effect in which increasing lasers powers led to more SGC5 internalization (**Figures 2B–D** show the results of a single laser power titration experiment). Basal epithelial cells in corneas injected with either SGC5 (green-fluorescing) or another lipophilic dye, FM4-64 (red-fluorescing) (29) responded identically to high power laser scanning (**Figure 2A**).

**Figure 4:**
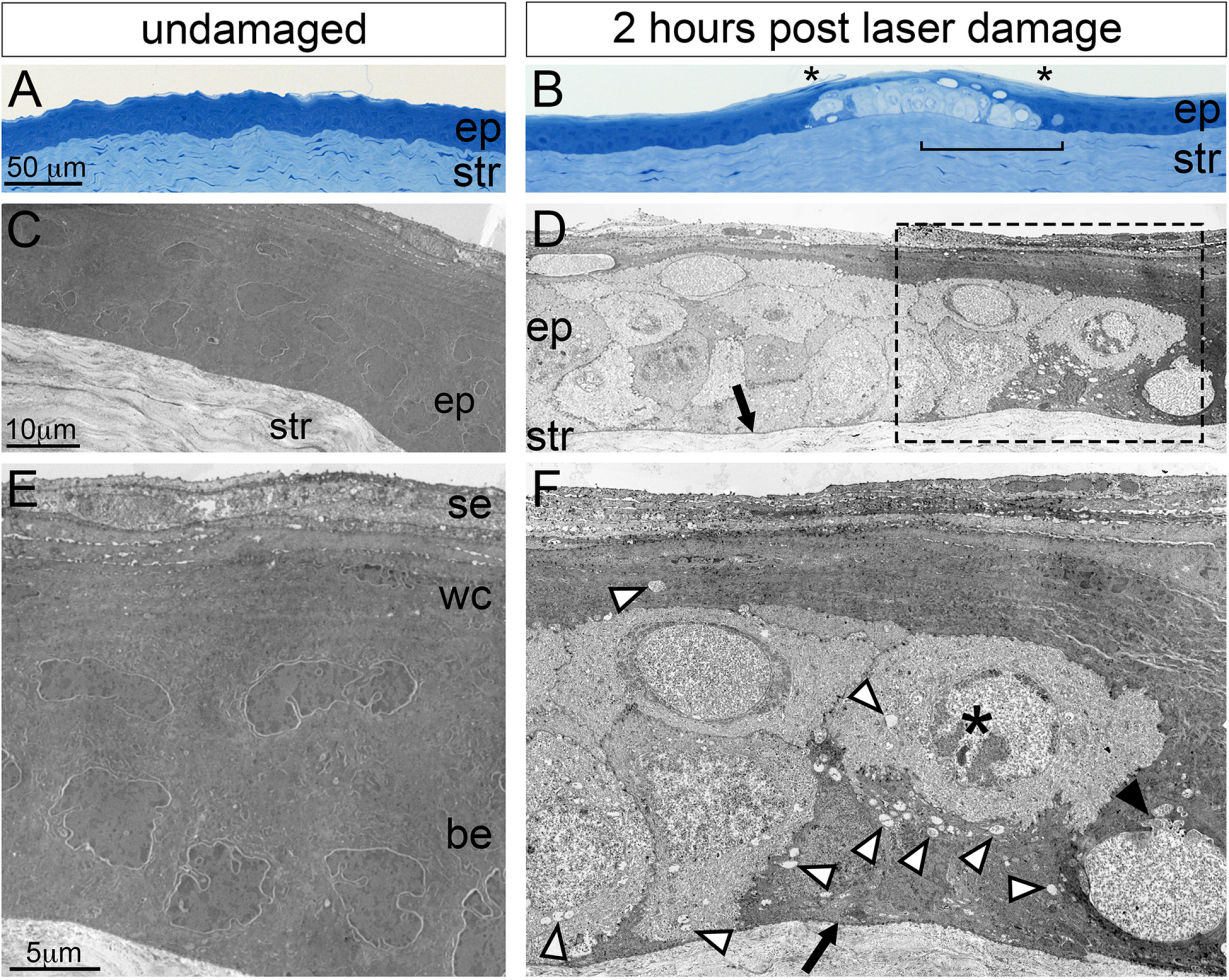
Histological analysis of corneal microdamage by light and transmission electron microscopy. Light microscopy imaging of toluidine blue stained Epon sections showing the corneal epithelial (ep) and stromal (str) layers in **(A)** undamaged and **(B)** microdamaged corneas. Damaged basal epithelium in **(B)** is bounded by asterisks characterized of the cytoplasm. The bracketed region in **(B)** corresponds to a similar region in panel **(D)**. **(C, D)** Transmission electron micrographs showing the corneal epithelium (ep) and stroma of **(C)** undamaged and **(D)** microdamaged corneas. Higher magnification showing the corneal epithelium **(E, F)**. The arrows in **(D, F)** indicates an intact basement membrane. The white arrowheads in **(F)** point to vacuoles, the black arrowhead in **(F)** points to a disrupted nuclear membrane, the “*” indicates a nucleus with bleached, disrupted morphology. se, superficial epithelium; wc, wing cell regions; be, basal epithelium.

As cell swelling is one of the hallmark features of necrotic death (31–33), we wanted to determine whether another feature of necrosis, early plasma membrane rupture (31–33), was observed following high power laser exposure. To examine this, eyes were co-injected with the hydrophilic dye, Alexa 647 hydrazide. Like SGC5, Alexa 647 hydrazide efficiently entered the stromal layers (data not shown), and surrounded epithelial cells but did not penetrate into the cytoplasm when imaged at low laser power (**Figures 2C, D**, **4**). When a region of the basal epithelial layer was scanned for 1 minute with the 488 nm laser set to 205 μW or higher, Alexa 647 hydrazide dye uptake in a subset of the damaged cells was observed (**Figures 2B, C**). While Alexa 647 hydrazide dye uptake was observed in 1 or 2 cells in regions scanned at 205 μW and 287 μW, a much larger proportion of cells were filled with Alexa 647 in regions scanned with a laser power of 528 μW. In contrast to SGC5 dye internalization, which was visible immediately after the completion of the microdamage protocol and increased linearly in all the cells within the scanned region over a one-hour period, Alexa 647 hydrazide appeared to fill the entire cells (cytoplasm and nucleus) and was detected in cells in an ‘all or none’ manner beginning 20 minutes after laser exposure (**Figure 2D**). In general, Alexa 647 uptake was not evident at the peak of cell swelling (**Figure 2D**, red arrowheads) and appeared as soon as cells began to contract. Together, these observations suggest that a brief period of high power laser scanning leads to cellular damage and necrosis of basal epithelial cells.

To further examine the extent of cell damage in corneas exposed to confocal laser stimulation we examined them histologically using brightfield and transmission electron microscopy (TEM). Brightfield imaging of toluidine blue-stained corneal sections (**Figures 4A, B**) revealed cellular and nuclear expansion, large empty intra- and extracellular vacuoles, and the bleaching of the cytosolic staining in both the basal epithelium and wing cell region while the overlying superficial epithelium was relatively intact. Examination of damaged corneal regions by TEM revealed nuclear fragmentation, chromatin condensation, bleached (i.e. less electron dense) cytosol, and the lack of cellular organelles (such as ER and mitochondria) in the basal cell and wing cell layers. The basement membrane adjacent to the damaged basal cell layer appeared intact (arrows in **Figures 4D, F**). Together, these histological data indicate that 1-minute of laser scanning of SGC5 membrane-loaded basal epithelium leads to rapid and selective cell necrosis in the basal epithelium as well as the overlying wing cells.

**Figure 5:**
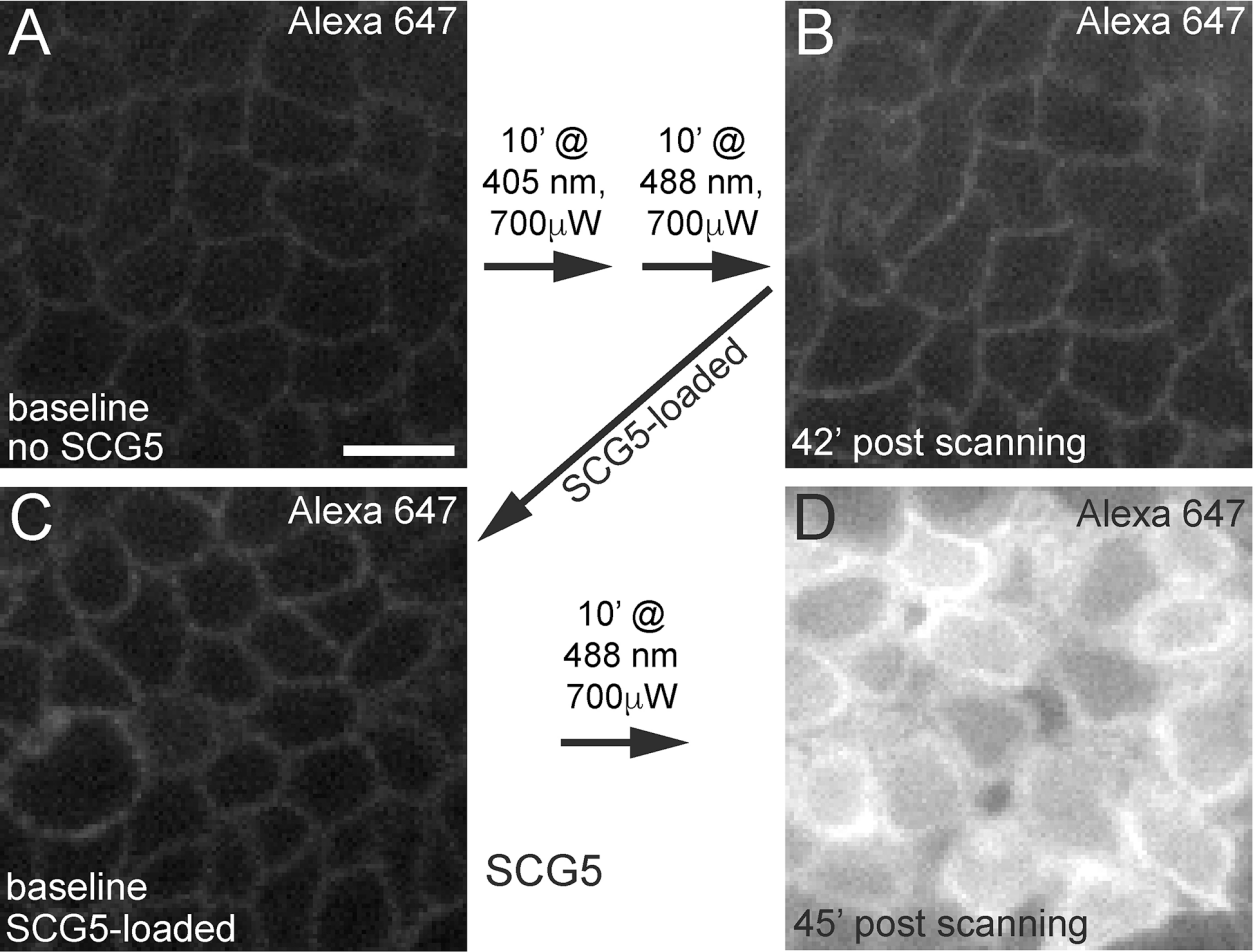
Confocal laser scanning-induced corneal microdamage is dependent on SCG5. Alexa 647 hydrazide injected eyes in the absence **(A, B)** or presence **(C, D)** of SCG5 were subjected to the wavelengths, laser powers and durations indicated on the arrows. At all other times, imaging was performed using the 640 nm laser line set at <5% laser power. **(B)** Scanning for 10’ at 405 nm (80% laser power setting, 225 μW) followed by another 10’ at 488 nm (80% laser power setting, 700 μW) did not lead to robust Alexa 647 hydrazide uptake or cell swelling 42’ after high power scanning. **(D)** Following SCG5 injection and scanning for 10’ at 488 nm (700 μW), robust Alexa 647 hydrazide uptake was observed. Scale bar = 10µm.

As fluorophore photoexcitation can lead to the production of cytotoxic reactive oxygen species (ROS) (34), we next wanted to determine whether the cellular damage observed following laser stimulation of the basal epithelium was directly caused by confocal laser exposure and/or was dependent on the SCG5 dye. To test this, we injected the anterior chamber with only Alexa 647 hydrazide and exposed the basal epithelium to high powered 405 nm and 488 nm laser stimulation. After exposing a 40 μm x 40 μm region of the basal epithelium to 10 minutes of exposure at 405 nm (~220 μW) followed immediately by another 10 minutes at 488 nm (700 μW), we did not observe any Alexa 647 cellular uptake, even up to 42’ minutes after stimulation (**Figures 5A, B**). However, after 1mM SGC5 was injected to the same cornea, followed by stimulation of the basal epithelium for 10 minutes at 488 nm (700 μW) (in a different region of the same cornea), robust Alexa 647 uptake was observed (**Figures 5C, D**). These findings demonstrate that cell damage following laser stimulation of the basal epithelium is dependent on the presence of the membrane lipophilic dye SGC5 (and likely FM-4-64 since it is also lipophilic) possibly through the production of cytotoxic ROS following intense fluorophore photoexcitation.

### *Cx3cr1*:GFP+ resident corneal macrophages respond to laser-induced damage

**Figure 6:**
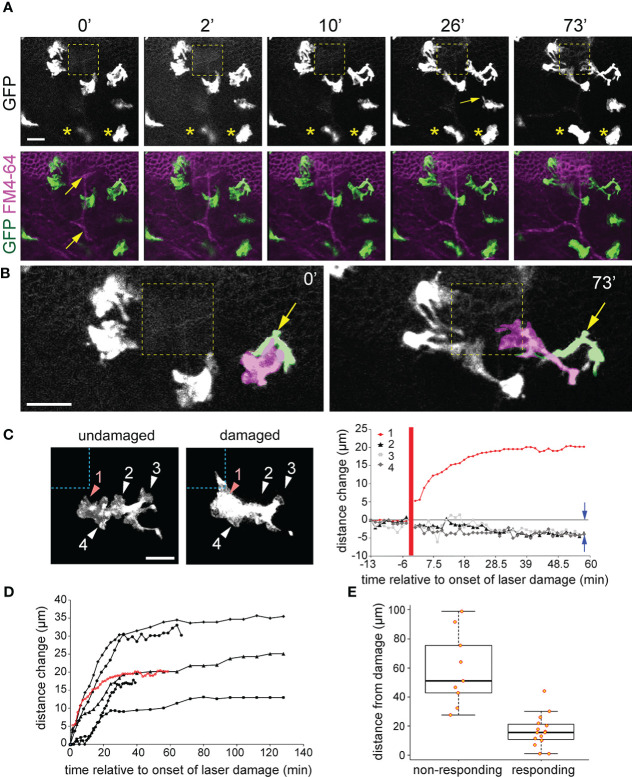
*Cx3cr1*:GFP+ macrophage response to basal epithelial microdamage. **(A)** When nearby FM4-64 labelled basal epithelium is damaged using high power confocal laser scanning, *Cx3cr1*:GFP+ macrophages project pseudopodia-like processes toward the damaged region (yellow dashed square). Times represent minutes after offset of high-powered laser exposure. The panel directly below **(A)** is a maximum intensity project along the z axis showing labelling of GFP (green) and FM4-64 (magenta). Yellow arrows indicate anterior stromal nerve fibers. **(B)** Zoomed-in regions from **(A)** at times 0’ and 73’. Two overlapping GFP+ cells, one a macrophage (shaded magenta) and the other a dendritic cell (shaded green and labeled with yellow arrow) defined by its long and slender morphology are observed. By 73’, the blue macrophage becomes highly polarized and projects to the damaged region while the green dendritic cell only extends slightly toward the damaged region. The remaining cells in **(B)** are all macrophages. **(C)** Imaging of a single macrophage before and after basal cell microdamage (indicated by the blue dashed box). The distance change over time of pseudopodia-like processes #1-4 is plotted on the right. Positive values indicate a change in length away from the cell and negative values indicate retraction. Data from the macrophage shown in **(A)** reveals that the pseudopodia closest to the damaged area (process 1) reached full extension after 30 minutes, and pseudopodia #2-4 which were further away from the microdamage retracted by approximately 5 µm as indicated by the blue arrows in the graph. The thick vertical red line indicates the time of injury. **(D)** Similar plot as in **(C)** showing the change in length of pseudopodia-like projections from six different macrophages that exhibited a response to epithelial microdamage. **(E)** Graph showing distance of “*responding*” and “*non-responding*” macrophages (see Methods for description) from the damage site; 16.8 ± 11.1 µm (responding) vs. 59.0 ± 25.3 µm (non-responding), P < 0.01, two tailed unpaired T test, n = 4 mice). Scale bar = 20 µm **(A, C)**, 30 µm **(B)**.

Given our ability to live-image and damage a small well-defined region of the corneal basal epithelium, we were next curious as to whether a leukocyte response to the damage could be observed. We focused our analysis on the resident population of stromal-localized corneal macrophages (RCSMs) (35–37) that can be readily visualized in *Cx3cr1^+/GFP^* mice (26, 38–41), and have been shown to participate in corneal wound healing (42–44). Live imaging of undamaged corneas from *Cx3cr1^+/GFP^* mice in whole eye preparations revealed stationary *Cx3cr1*:GFP+ RCSMs in the central-most region with pleomorphic morphology, often appearing circular or oblong in shape (**Figure 6**; **Supplemental Movies 1**, **2**). GFP+ RCSMs had pseudopodia-like extensions that were often semi-circular in shape and ~5 μm in diameter and cycled between an extended and retracted state approximately every 2-3 minutes. Although *Cx3cr1*:GFP+ cells with dendritic cell morphology were frequent in the corneal periphery (data not shown), we only observed them in the central cornea on rare occasions (**Figure 6B**, green-shaded cell labeled with arrow), consistent with previous findings (45).

**Figure 7:**
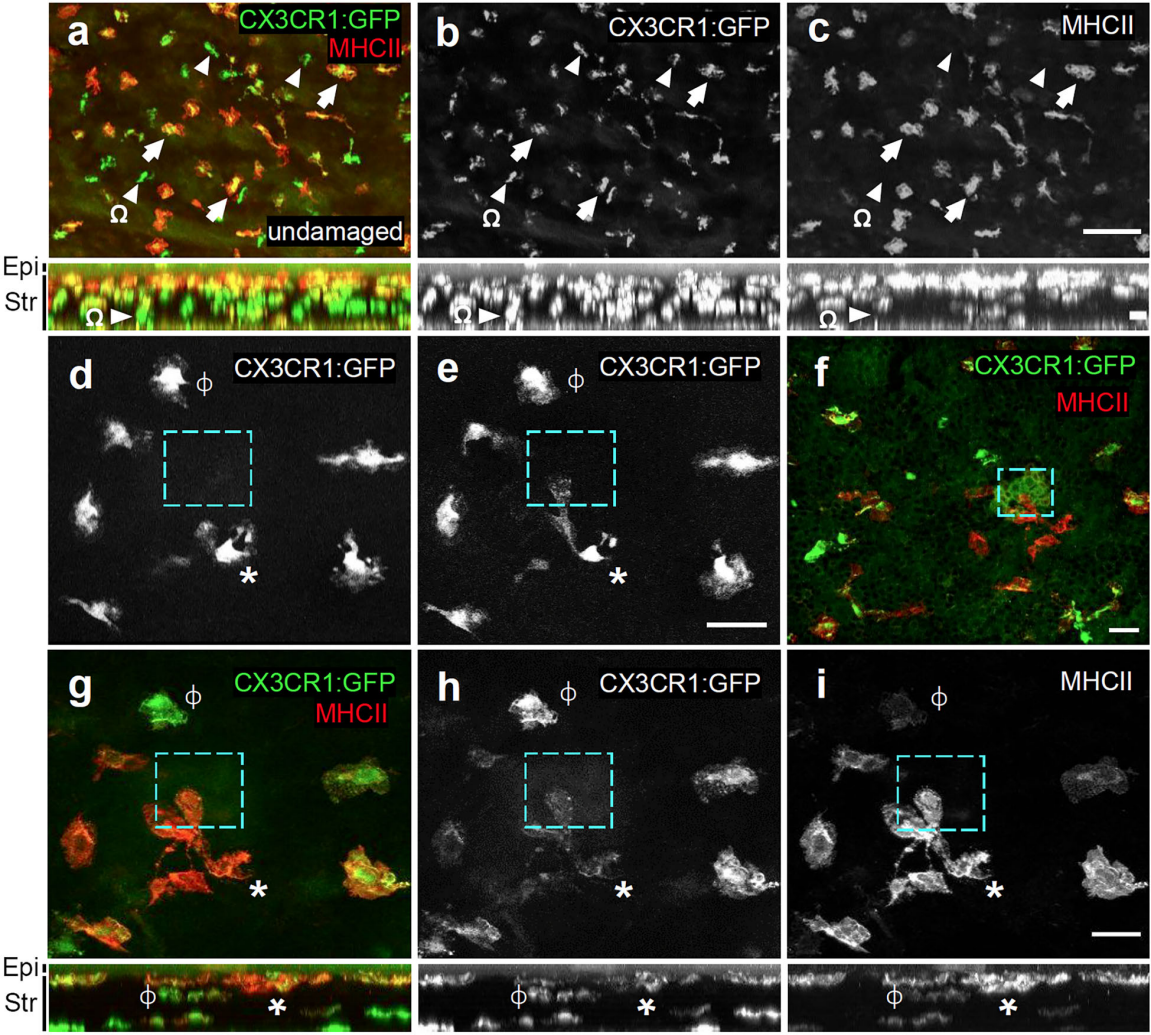
Responding cells co-label with MHCII. **(A–C)** Flat-mount of a PFA-fixed undamaged *Cx3cr1^GFP/+^* cornea immunolabelled for MHC class II (red) and GFP. Not all GFP+ cells are MHCII+ (arrows indicate GFP+MHCII+ double-positive cells; arrowheads = GFP+ only). Stratification of MHCII+ cells toward apical region of the stroma is observed. **(D, E)** Live imaging of *Cx3cr1*:GFP+ macrophages before **(D)**, and 70’ after microdamage **(E)**. The cyan dashed box represents the region of microdamage. “*” marks a macrophage that has become polarized and has extended a pseudopod-like process toward the region of damage. ϕ marks a non-responding macrophage. **(F–I)** Immunolabeling of the cornea from **(E)** that was fixed in 4% PFA and labelled for MHC class II+ (red) and GFP. In **(G–I)**, ϕ and * indicate the same cells as in **(D, E)**. The panels directly below **(A–C)** and **(G–I)** represent their corresponding X-Z projections. Epi, epithelium; Str, stroma. Cyan box = microdamaged area. The responding cell (*) co-labels with MHCII. Scale bars represents 100μm **(A–C)** 30μm **(D–I)**.

Enucleated eyes from *Cx3cr1^+/GFP^* mice were subjected to the laser-microdamage technique described above and Z-stack imaging was performed through the basal epithelial and stromal layers. *Cx3cr1*:GFP+ RCSMs closest to the site of damage responded by extending usually one or sometimes two broad pseudopodia-like projections towards the damaged region (**Figures 6**, **7**; **Supplemental Movies 3**, **5**. All of the eyes examined, n=7 (all from different mice), exhibited a RCSM response which, as defined in the Methods, is any cell in which at least one pseudopodia-like projection exhibited a sustained increase in length of greater than 10 μm towards the site of damage as measured 1-hour after damage protocol was performed. Pseudopodia closest to the damaged region grew markedly in length (up to 30 μm), while the processes on the same macrophage, further away from the damaged site retracted (**Figure 6C**), leaving the cell polarized. RCSMs began responding 5-10 minutes after the initiation of laser-damage protocol and full extension of processes was reached by 30-40 minutes (**Figures 6C, D**), after which no further morphological changes were observed. There was no observable migration of RCSM cell bodies toward the site of damage in any of the axes up to 2.5 hours after microdamage induction (data not shown). Within a given field of view, macrophages located within 30 μm (along the x-y axis) from the damage site tended to exhibited some form of morphological response (minimally some form of process extension) toward the site of damage compared to those beyond that distance (Fig 6E; mean distances from damage site: 16.8 ± 11.1 µm (“responding”) vs. 59.0 ± 25.3 µm (“non-responding”), P < 0.01, two tailed unpaired T test, n = 4 corneas from 4 different mice). In summary, following basal cell damage, nearby resident corneal macrophages extend pseudopodia that are positioned closest to the damage and retract pseudopodia that are further away thus becoming polarized.

Although more abundant in the periphery, putative *Cx3cr1*:GFP+ dendritic cell morphology, defined by the slender processes morphology, were occasionally observed in the central cornea. When positioned adjacent to a site of microinjury, they maintained their morphology and did not adopt the same highly polarized morphology as neighboring RCSMs (**Figure 6B** cell shaded pink, **Supplemental Movie 4**). They did, however, show some subtle morphological changes in response to microdamage, typically through the extension of one or two <10 μm long filipodia-like processes from an existing filipodia branch (**Supplemental Movie 4**).

As corneal macrophages are heterogeneous with respect to marker gene expression and function (40, 43, 46), we next wanted to determine what macrophage subtype was responding to the laser-induced basal epithelial cell damage. We live-imaged and then retrospectively immunolabelled the laser-damaged corneas of *Cx3cr1^+/GFP^* mice with MHC class II, which is expressed in a subset of macrophages in the anterior region of the stroma (40, 45). Z-X projections confirmed that MHC class II labelling was most abundant in *Cx3cr1*:GFP+ cells in the anterior part of the stroma closest to the basal epithelium, and that *Cx3cr1*:GFP+ cells posterior to these were mostly MHC class II- (**Figure 7A**). *Cx3cr1*:GFP+ RCSMs responding to basal epithelial cell damage were located close to the basal epithelium and co-labelled with MHC class II (**Figures 7D–I**). These findings show that following basal epithelial cell damage, MHC class II+, *Cx3cr1*:GFP+ RCSMs are able to respond in a robust and rapid manner by extending pseudopodia-like processes towards the site of injury.

## Discussion

In this study, we developed an approach to simultaneously image the cornea with high cellular resolution and induce precisely localize corneal epithelial microdamage in a live, whole-eye preparation. Based on the rapid cell swelling, vacuolization and cytoplasmic bleaching, cells in the damaged region appear to undergo necrosis. This is also supported by the cytoplasmic and nuclear uptake of the membrane impermeant Alexa 647 hydrazide which suggests rupturing of both the plasma and nuclear membranes which are hallmarks features of necrosis. We used this microdamage paradigm to visualize the real-time response of nearby resident stromal *Cx3cr1*:GFP+ RCSMs. RCSMs responding to basal epithelial damage became polarized through the extension of pseudopodia-like processes towards the damage site within 5-10 minutes after the initiation of damage. Our study adds to the existing body of literature which has utilized live-cell imaging *in vivo* (24, 25) or in culture (21–23) to visual corneal leukocytes responses to external stimuli and damage paradigms.

The dependency of our microdamage procedure on the presence of the lipophilic dyes, SGC5 or FM4-64, suggests that cytotoxic ROS generated by dye photoexcitation mediate cell damage/necrosis. It is established that fluorophore excitation can lead to the formation of ﻿singlet oxygen and superoxide radicals that damage proteins and cellular structures including membranes, and can lead to cytotoxicity (47–51). Furthermore, the constant replenishment of SGC5 or FM4-64 into cellular membranes due to their high concentration in the anterior chamber may make cells especially vulnerable to ROS-mediated cell damage when imaged continuously at high laser power. Regardless of the mechanism, our data emphasize the importance of titrating laser power settings when performing live imaging experiments using fluorophores, especially if they are lipophilic, to prevent unwanted cytotoxicity.

Our ability to live-image and damage a precise region of the corneal epithelium made it possible to follow the real-time response of RCSMs in *Cx3cr1^+/GFP^* mice. *Cx3cr1*:GFP+ RCSMs have been described previously (38, 40, 41, 46, 52) and have been examined using live-cell imaging (24, 25). Previous work by others have shown that the motility and behavior of *Cx3cr1*:GFP+ cells using intravital multiphoton microscopy in response to corneal thermal cautery burn was minimal (25), however, this study was performed on *Cx3cr1^GFP/GFP^* homozygous mice, so it is unclear whether the lack of a robust motility phenotype was due to *Cx3cr1* deficiency. While we did not observe RCSM lateral motility following epithelial microdamage in our study, we cannot rule out the possibility that this was due to performing our experiments at room temperature which has been shown to affect motility of corneal CD11c+ dendritic cells and major histocompatibility complex class II (MHC)-II+ mature antigen-presenting cell populations (25). Although we observed subtle changes in dendritic cell morphology in response to epithelial microdamage, this cell type was not the focus of our study and it will be interesting to study their response in detail in the future.

In the undamaged corneas, the constant extension and retraction of short pseudopodia-like projections in *Cx3cr1*:GFP+ RCSMs was reminiscent of the baseline/steady state sampling behavior of dendritic cells previously described (21, 25). We propose that individual RCSM projections function as independent surveyors of the local corneal environment, and that upon detection of an environmental damage cue transduce signals that negative regulate the stability of neighboring projections on the same cell that are more distal to the cue, thus enabling the re-distribution of plasma membrane to the elongating projection. The drastic change in RCSM cell polarity and formation of pseudopodia-like extensions in our study is very similar to that observed in brain microglia (53–55), and in ﻿peritoneal wall resident tissue macrophages (56), which in both cases, continuously extend and retract processes, and in response to local cell damage extend processes toward the damaged region within 30 minutes (53–56). Our experimental approach represents a simple system to visualize and study the mechanism of these general resident tissue macrophage and microglial responses.

The mechanism that mediates resident RCSM pseudopodia extension towards the damage site remains to be determined, however, given that basal epithelial cell membranes are ruptured following our damage protocol, it is likely that released cellular contents are involved in initiating the macrophage response. This would be consistent with our observation that only macrophages within 30 μm of the site of microinjury tended to respond to the damage. Nucleotides, such as ATP, are good candidates for this function as they are among the earliest molecules released from damaged cells (57). Furthermore, ATP (presumable released from damaged cells) has been shown to mediate the rapid responses of brain microglial (53), and peritoneal wall resident tissue macrophages (56) to local cellular damage.

Eliciting an appropriate wound healing response to match the type and degree of cellular damage is especially important in a tissue such as the cornea, which must maintain its transparency for optimal vision. Macrophage heterogeneity and asymmetric distribution in the cornea may play an important role in this process. It has previously been suggested that the more anterior positioned MHCII+ stromal macrophages function in the innate response to penetrating antigens, while deeper MHCII- macrophages may function in a barrier capacity by responding directly to bacterial threats and preventing penetration of pathogens or physiologic wound healing (45). Our study suggests that *Cx3cr1*:GFP+/MHC class II+ resident stromal macrophages play an active role in surveillance and have the capacity to function as first responders to corneal epithelial cell damage, even to a small, benign microinjury of the basal epithelium. Interestingly, we observed an instance in which a more distally positioned RCSM appeared to initiate a projection toward the site of microinjury but quickly retracted it (**Figure 6A**, arrow). Although it is possible that this aborted pseudopodial projection may simply be the consequence of a low concentration of a diffusible molecule coming from the damage site, the possibility that responding RCSMs closer to the damage may actively inhibit RCSMs that are further away cannot be ruled out.

Although the biological relevance of the RCSM responses we observed to basal cell microdamage is unclear, recent work on peritoneal wall resident tissue macrophages may provide some insight (56). Like our findings, in response to nearby tissue microdamage, these macrophages extend pseudopodia-like processes which surround the damaged area. This is thought to sequester the region of damage and act like a “cloak” to prevent feedforward signaling, downstream infiltration of neutrophils and subsequent inflammatory damage (56). It remains to be determined whether such a mechanism also occurs in the cornea. However, it is a reasonable possibility given that the corneal epithelium is continuously subjected to many different forms of insult and damage, and prevention of unnecessary and potentially damaging inflammatory responses is desired.

## Data availability statement

The original contributions presented in the study are included in the article/**Supplementary Material**, further inquiries can be directed to the corresponding author/s.

## Ethics statement

The animal study was reviewed and approved by the University of Victoria, Animal Care Committee in accordance with guidelines set by the Canadian Council for Animal Care.

## Author contributions

SG: preformed all experiments, contributed to experimental design, and co-wrote manuscript; BG: performed transmission electron microscopy and toluidine blue staining AL: co-designed initial lipophilic dye injection and imaging protocol; KD: co-designed initial lipophilic dye injection and imaging protocol; RC: co-designed initial lipophilic dye injection and imaging protocol, contributed to experimental design, and co-wrote manuscript. All authors contributed to the article and approved the submitted version.
